# A multi-omics dataset of human transcriptome and proteome stable reference

**DOI:** 10.1038/s41597-023-02359-w

**Published:** 2023-07-13

**Authors:** Shaohua Lu, Hong Lu, Tingkai Zheng, Huiming Yuan, Hongli Du, Youhe Gao, Yongtao Liu, Xuanzhen Pan, Wenlu Zhang, Shuying Fu, Zhenghua Sun, Jingjie Jin, Qing-Yu He, Yang Chen, Gong Zhang

**Affiliations:** 1 Key Laboratory of Functional Protein Research of Guangdong Higher Education Institutes and MOE Key Laboratory of Tumor Molecular Biology, Institute of Life and Health Engineering, Jinan University, Guangzhou, China; 2 Sino-French Hoffmann Institute, School of Basic Medical Sciences, State Key Laboratory of Respiratory Disease, Guangzhou Medical University, Guangzhou, China; 3 CAS Key Laboratory of Separation Science for Analytical Chemistry, National Chromatographic Research and Analysis Center, Dalian Institute of Chemical Physics, Chinese Academy of Science, Dalian, China; 4 School of Biology and Biological Engineering, South China University of Technology, Guangzhou, China; 5 Department of Biochemistry and Molecular Biology, Beijing Key Laboratory of Gene Engineering Drug and Biotechnology, Beijing Normal University, Beijing, China

**Keywords:** Transcriptomics, Proteomics, Translation

## Abstract

The development of high-throughput omics technology has greatly promoted the development of biomedicine. However, the poor reproducibility of omics techniques limits their application. It is necessary to use standard reference materials of complex RNAs or proteins to test and calibrate the accuracy and reproducibility of omics workflows. The transcriptome and proteome of most cell lines shift during culturing, which limits their applicability as standard samples. In this study, we demonstrated that the human hepatocellular cell line MHCC97H has a very stable transcriptome (*r* = 0.983~0.997) and proteome (*r* = 0.966~0.988 for data-dependent acquisition, *r* = 0.970~0.994 for data-independent acquisition) after 9 subculturing generations, which allows this steady standard sample to be consistently produced on an industrial scale in long term. Moreover, this stability was maintained across labs and platforms. In sum, our study provides omics standard reference material and reference datasets for transcriptomic and proteomics research. This helps to further standardize the workflow and data quality of omics techniques and thus promotes the application of omics technology in precision medicine.

## Background & Summary

The booming applications of omics technologies provide unprecedented insights into biology and medicine. However, the reproducibility of omics technology has been questioned for a long time. A study in genomic sequencing showed zero sensitivity in finding pathogenic mutations using whole exome sequencing of 57 patients^1^. The mutation of 40 circulating tumor DNA (ctDNA) samples, sequenced by two individual companies, showed only 12% congruence^2^. In the field of RNA-seq, a wide variety of methodologies limits reproducibility^3^. In the field of proteomics, a study of Human Proteome Organization (HUPO) tested 20 highly purified recombinant human protein samples. Each protein contains one or more 1250 Da unique pancreatic peptides. The samples were then distributed to 27 laboratories for identification. The results found that only 7 of 27 laboratories reported all 20 proteins, whereas only 1 laboratory reported all 1250 Da trypsin peptides^4^. Another study found that even using optimal conditions and a uniform standard operating procedure, the median value of protein repeatability for the same mixture sample in different laboratories was only 75%^5^. In addition, the repeatability of multiple quantitative tests on the same sample in the same laboratory and between different laboratories is less than 80%^6^. Other studies show that omics research’s lack of reliability and repeatability is one of the biggest obstacles to narrowing the gap between personalized medicine research and practice^7,8^.

Reference materials are needed to examine the omics workflow’s accuracy and reproducibility. For genome (DNA) sequencing, reference genome samples, e.g., ΦX174 viral DNA and NA12878 human cell line genomic DNA, were widely used as standards. Mixed genomic standards are used for variant calling benchmarks^9^. DNA standards are easy to produce because of the high fidelity of DNA replication of normal genomes. However, the transcriptome (RNA) and proteome are qualitatively and quantitatively highly variable due to various kinds of factors, including intrinsic factors (e.g., senescence, cell cycles, contact inhibition, etc.) and extrinsic factors (e.g., temperature, osmolarity, buffer content, oxidation, etc.), which set challenges on making reference standard transcriptome and proteome sample. Since 2004, the Universal Human Reference RNA^10^ has been commercialized as a “standard” RNA sample for RNA-seq benchmarking^11–15^. However, the RNA content of the Universal Human Reference RNA sample may shift during long-time production. Universal Human Reference RNA is a pooled RNA of 10 cancerous cell lines, including Hela, which is well known for its instability^16–21^. Indeed, in the lot-to-lot comparison of the Universal Human Reference RNA, Pearson’s correlation coefficient reached only 0.9736^10^, which demonstrated such instability even in a short production period. Such variation, which is expected to multiply during a long production period, is not sufficient to evaluate the reproducibility of the advancing next-generation sequencing techniques with increasing depth and resolution. The proteome is more variable than the transcriptome due to the massive translational regulation^22^. Therefore, a stable proteome reference is more difficult to produce. Since 2003, the Proteomics Standards Initiative standardized the data formats of mass spectrometry (MS)-based proteomics but did not plan to provide a human proteome standard sample^23,24^. Till 2021, proteome standard material has not been considered in the quality standards in research facilities^25^. Currently, a mixture of 18~48 recombinant proteins is used as a “test standard” or spike-in for proteomics^5,26–29^. However, such a small number of proteins could hardly form a reference standard for complex proteome samples.

**Fig. 1:**
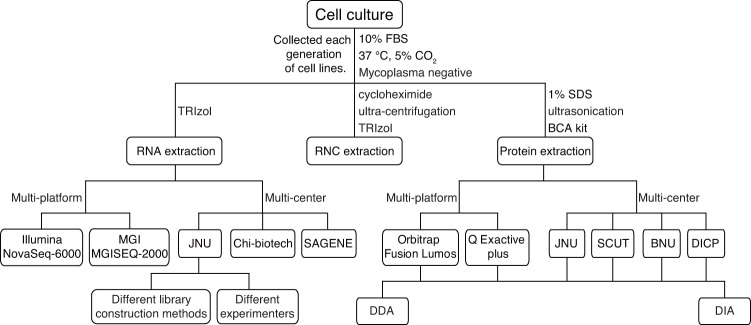
Workflow of sample preparation and omics dataset generation.

In this study, we collected the 8~12 generation cells of 5 cell lines, A549, HCCLM3, HCCLM6, Hela, and MHCC97H. Their RNA was extracted to generate a transcriptome sequencing dataset (Fig. 1). Quantitative analysis results showed that MHCC97H has high stability in the transcriptome. Subsequently, we generated the MHCC97H translatome sequencing dataset and protein mass spectrometry dataset. In the comprehensive tests of multiple laboratories and multiple platforms, MHCC97H showed high stability in both transcriptome and proteome. In conclusion, we demonstrate that the MHCC97H cell line has a stable transcriptome and proteome that can be used as an omics standard to evaluate and calibrate omics workflows. We also provided transcriptome datasets of multiple cell lines, as well as MHCC97H translatome and mass spectrometry datasets, which provide a reliable reference for the quality control of omics data.

## Methods

### Cell culture and materials

Hela and A549 cell lines were purchased from American Type Culture Collections (ATCC, Rockville, MD, USA) and authenticated by short tandem repeat profiling. The human hepatoma MHCC97H, HCCLM3, and HCCLM6 cells were provided by Professor Yinkun Liu, Fudan University^12^. In fact, MHCC97H, HCCLM3, and HCCLM6 cell lines are derived from the parent MHCC97 cell, and their potential metastatic ability increased successively^30^. The MHCC97H cell line was isolated from the parent MHCC97 cell with high metastatic potential^31^. HCCLM3 was derived from MHCC97H and was characterized by high lung metastasis^32^. HCCLM6 was also derived from the parent MHCC97H cell, and was characterized by high lung metastasis and lymphatic metastasis^33^ MHCC97H, HCCLM3, and HCCLM6 cell lines were typical hepatocellular carcinoma cell lines, and their characteristics, phenotypes and representative disease characteristics have been reported in detail^30^. All cells were detected free of mycoplasma during maintenance and upon experiments. These cells were cultured in a DMEM medium with 10% FBS and 1% penicillin/streptomycin. Culture conditions for all cell lines were 37 °C, 5% CO_2_.

### Detection of mycoplasma contamination

We used the Mycoplasma Detection Kit (ExCellBio, MB000-1591, China) to detect cell contamination by mycoplasma. Detailed operation steps were as follows: 1~1.5 mL cell culture supernatant was put into a centrifuge tube and centrifuged at 13000 rpm for 5 min. Then the supernatant was discarded and the precipitate was washed once with PBS. 100 µL of lysate was added to lyse the cells, inverted upside down to mix well, and left at room temperature for 5 min. The cell lysate was then incubated at 95 °C for 5 min, centrifuged at 13000 rpm for 5 min, and the supernatant was transferred to a new centrifuge tube. The PCR reaction was carried out with toke 1~2 µL supernatant as a template, and the amplified products were electrophoresis by 2% agarose gel.

### RNA extraction

Each generation of cells was cultured to 80~90% confluency, then washed twice with RNase-free PBS (LEAGENE, IH0142, China), and then isolated by using TRIzol RNA extraction reagent (Invitrogen, 15596026, USA), detailed steps were as follows: Cells were collected in 1.5 mL EP tubes, centrifuged at 230 × *g* for 3 min, the supernatant was discarded and PBS was added to wash the cells. Then added 1 mL of TRIzol into a fume hood, mixed well, and placed at room temperature for 5 min, and added 200 µL chloroform and the mixture was vortexed vigorously for 15 s and placed at room temperature for 3 min. After centrifugation at 12000 × *g* at 4 °C for 15 min, the sample was divided into three layers (RNA in the upper aqueous phase), and the upper aqueous phase was sucked into a new EP tube. Then added 800 µL isopropyl alcohol, inverted and mixed, and placed at - 20 °C overnight. The next day, centrifuged at 12000 × *g* at 4 °C for 30 min, and removed the supernatant. Added 1 mL of 75% ethanol to wash the RNA precipitation (precooled at - 20 °C), centrifuged at 7500 × *g* at 4 °C for 5 min, and removed the supernatant, repeat this step once again. Used 20 µL of RNAase-free water to redissolve the pellets, and ran electrophoresis with 1% agarose gel after measuring RNA concentration.

### Ribosome-nascent chain complex-RNA (RNC-RNA) extraction

The RNC extraction was performed as we previously reported^34^. In brief, cells from each generation were cultured to 80~90% confluence, pretreated with 100 mg/mL cycloheximide for 15 min, then precooled PBS was washed and 2 mL of cell lysis buffer (1% Triton X-100, 20 mM HEPES-KOH (pH 7.4), 15 mM MgCl_2_, 200 mM KCl, 100 mg/mL cycloheximide and 2 mM dithiothreitol (DTT) (Solarbio, D8220, China)) was added. After 30 min ice bath, cell lysates were scraped and transferred to 1.5 mL RNase-free tubes. Cell debris were removed by centrifuging at 16200 × *g* for 10 min at 4 °C. Supernatants were transferred on the surface of 20 mL of sucrose buffer (30% sucrose, 20 mM HEPES-KOH (pH 7.4), 15 mM MgCl_2_, 200 mM KCl, 100 mg/mL cycloheximide and 2 mM DTT). RNC was pelleted per ultra-centrifugation at 42500 rpm for 5 h at 4 °C. Subsequently, RNC-RNA was extracted from RNC particles using TRIzol RNA extraction reagent following the manufacturer’s instructions.

### mRNA-seq and RNC-seq

For all mRNA-seq and RNC-seq, DNase I (Thermo Fisher Scientific, EN0525, USA) treatment was performed prior to the RNA library construction to remove DNA contamination according to the manufacturer’s instructions. For mRNA-seq, our study used two methods to construct the sequencing library, including Oligo-dT method (PolyA + mRNA strategy) and the rRNA depletion method (Ribominus strategies).

We used Library Preparation VATHS mRNA Capture Beads (Vazyme, N401-02, China) to purify polyA + mRNA from total RNA. Then, according to different experimental designs, used the MGIEasy RNA Library Prep Kit (MGI, A0210, China) for MGI platform or VAHTS Universal V6 RNA-seq Library Prep Kit (Vazyme, NR604-01, China) for Illumina platform to constructed the sequencing libraries for Oligo-dT method, according to each manufacturer’s instruction. Before the rRNA depletion sequencing libraries were constructed, rRNA was removed from total RNA by probe hybridization followed by RNase H degradation as we previously reported^35,36^. And the rRNA depletion sequencing libraries were also constructed by using the MGIEasy RNA Library Prep Kit according to the manufacturer’s instructions too.

For RNC-seq, only Oligo-dT method was used for library construction, which was the same as mRNA-seq. Among all the mRNA-seq and RNC-seq data involved in this project, only the mRNA library construct by Sagene Co. Ltd. was sequenced by NovaSeq-6000 platform (Illumina, China) for 300 cycles, and the rest were sequenced on a MGISEQ-2000 platform (MGI, China) for 210 cycles.

The high-quality reads were subjected to the subsequent bioinformatics analysis. The adapter sequences were trimmed from the reads. Then reads were mapped to transcripts using the hyper-accurate mapping algorithm FANSe3^37,38^ in the next-generation sequencing analysis platform “Chi-Cloud” (http://www.chi-biotech.com). Gene expression levels were quantified using the RPKM (reads per kilobase per million reads) method^39^. Genes with at least 10 reads were considered quantifiable genes^40^.

### RNA degradation

We tested the RNA samples under degradation conditions to investigate how the degradation affects their applicability to serve as a reference, and to test whether our procedure could tolerate the degradation and provide comparable results as the non-degraded counterparts.

For RNA samples that were slightly degraded during RNA extraction, we used Oligo-dT method and the rRNA depletion method for library construction. The probe sequences used in the rRNA depletion method were listed in supplementary Table 1. The library construction method for RNA-degraded samples treated with RNase A was as follows: We randomly selected a complete total RNA sample without degradation from the 1~9 generations of MHCC97H (Fig. 3a). For example, in the third generation, the concentration of extracted total RNA was 1299.16 ng/µL, the total volume was 20 µL, and 29 µg of total RNA was finally obtained. We performed a series of RNase A degradation experiments, each of which contained 2 µg total RNA of the third-generation cell line as starting material. 1 ng RNase A was added to each of the five experimental groups (except the non-degraded group), and then reacted for 30 s, 1 min, 2 min, 5 min, and 1 h, respectively. Finally, 0.5 U RNaseOUT (Thermo Fisher Scientific, 10777019, USA) was added for the termination reaction. All experiments were operated at room temperature. The library was constructed by the rRNA depletion method, and evaluated in the same way as described above (paragraph “mRNA-seq and RNC-seq”).

### Protein trypsin digestion

Each generation of cells was cultured to 80~90% confluency, then treated with 0.25% trypsin-EDTA (Gibco, 25200056, USA), centrifuged at 300 × *g* for 5 min at room temperature, washed twice with PBS, and the supernatant was removed by centrifuge. Cells were dissolved in 1% SDS lysis buffer (Beyotime, P0013G, China) and the protein concentration was measured by a BCA quantification kit (Thermo Fisher Scientific, 23227, USA). The protein digestion was performed by filter-aided sample preparation (FASP)^41^ method. In brief, protein samples were treated with 8 M urea (8 M urea in 0.1 M Tris-HCl, pH 8.5), resulting in a final concentration of urea ≥4 M. Next, an appropriate amount of DTT was added to a concentration of 50 mM and incubated at 37 °C for 1 h. Iodoacetamide solution (IAA) (Merck, I6125, USA) was added to a concentration of 120~150 mM, and incubated at room temperature for 30 min in the dark. Each solution was transferred into a 10 KDa ultrafiltration tube (Merck, UFC501096, USA) and centrifuged at 12000 × *g* for 15 min. The filter tube was washed twice with 8 M urea (200 µL each time) and then washed three times (200 µL each time) with 50 mM triethylammonium bicarbonate (TEAB) (Thermo Fisher Scientific, 90114, USA). Finally, trypsin (Promega, V5280, USA) was added at the ratio of 1:40 (trypsin: protein), and incubated at 37 °C overnight. After 16 hours, all peptides were collected by centrifugation at 12000 × *g* for 20 min. Then washed the filter tubes twice with 50 mM TEAB (200 µL each time), and all eluted peptides were collected and mixed. Their concentrations were determined using the Pierce Quantitative Fluorometric Peptide Assay kit (Thermo Fisher Scientific, 23290, USA). Finally, all peptides were lyophilized and stored at -80 °C.

The peptides were then redissolved using 200 µL 0.5% trifluoroacetic acid (TFA) (Macklin, T818782, China) solution and desalted using Waters C18 columns (Waters, WAT054955, USA). The procedure for desalting was as follows: the C18 columns were activated with 1 mL acetonitrile (ACN) (Thermo Fisher Scientific, A955-4, USA) and then equilibrated twice with 1 mL condition buffer (20% ACN with 0.1% TFA). All peptides were then loaded into the C18 columns and repeated 3 times. Then the C18 columns were washed 5 times with 1 mL of washing buffer (0.1% TFA). Finally, all peptides were eluted with elution buffer (70% ACN with 0.1% TFA), lyophilized, and stored at -80 °C.

### Data-dependent acquisition (DDA) mass spectrometry

For data-dependent acquisition analysis, data were collected by Q Exactive Plus (QE+) mass spectrometer equipped with EASY-nLC 1000 system (Thermo Fisher Scientific, USA) and Orbitrap Fusion Lumos mass spectrometer equipped with EASY-nLC 1200 system (Thermo Fisher Scientific, USA) respectively.

#### QE+ parameter setting

Each injection consisted of 2 µg of peptides and 1 µL of standard peptides (iRT peptides) (Biognosys, Ki-3002-2, Switzerland). The samples were separated by a 100 µm × 20 mm, 5 µm C18 nano trap column (Thermo Fisher Scientific, AAA-164564, USA) and a 75 µm × 250 mm, 2 µm C18 analytical column (Thermo Fisher Scientific, 164941, USA), respectively. In the analytical column, the samples were eluted at a flow rate of 300 nL/min for 120 min, and the elution gradient was: 3~7% solvent B, 4 min; 7~18% solvent B, 70 min; 18~25% solvent B, 20 min; 25~35% solvent B, 16 min; 35~40% solvent B, 1 min; 40~90% solvent B, 9 min (solvent A: 98% H_2_O, 2% ACN, 0.1% FA; solvent B: 98% ACN, 2% H_2_O, 0.1% FA). The parameters of the mass spectrum were set as follows: MS1 scan range: 400 to 1200 m/z, resolution: 70000, AGC (auto gain control) target: 3e6, max injection time: 60 ms. Top-20 parent ions of MS1 were selected for MS2 collection. MS2 scan resolution: 17500, isolation window: 1.6 m/z, HCD (higher collision energy dissociation): 32%, AGC target: 5e5, max injection time: 50 ms, NCE (normalized collision energy): 27%, dynamic exclusion: 30 s.

#### Orbitrap fusion lumos parameter setting

Each injection consisted of 2 µg of peptides and 1 µL of iRT peptides. The samples were separated by a 150 µm × 20 mm, 1.9 µm C18 nano trap column (homemade) and a 150 µm × 300 mm, 1.9 µm C18 analytical column (homemade), respectively. In the analytical column, the samples were eluted at a flow rate of 600 nL/min for 120 min, and the elution gradient was: 5~12% solvent B, 28 min; 12~24% solvent B, 58 min; 24~38% solvent B, 25 min; 38~95% solvent B, 1 min; 95% solvent B, 8 min. The parameters of the mass spectrum were set as follows: MS1 scan range: 350 to 1500 m/z, resolution: 120 k, AGC: 4e5, max injection time: 50 ms; MS2 scans resolution: 15 k, isolation window: 1.6 m/z, HCD: 31%, AGC target: 5e4, max injection time: 50 ms, cycle time: 3 s, dynamic exclusion: 30 s.

### Data-independent acquisition (DIA) mass spectrometry

The mass spectrometry data were collected using QE+ and Orbitrap Fusion Lumos for data-independent acquisition analysis, respectively.

#### QE+ parameter setting

Each injection consisted of 2 µg of peptides and 1 µL of iRT peptides. Samples were analyzed in the data-independent acquisition method. The liquid conditions were the same as in the data-dependent acquisition method mentioned above. The parameters of the mass spectrum were set as follows: MS1 resolution: 70000; MS2 resolution: 17500, m/z range: 400 to 1200 m/z, variable acquisition windows: 30, AGC target: 3e6, injection time: 60 ms, NCE: 27%, AGC target: 1e6, max injection time: auto.

#### Orbitrap fusion lumos parameter setting

Each injection consisted of 2 µg of peptides and 1 µL of iRT peptides. Samples were analyzed in the data-independent acquisition method. The liquid conditions were the same as in the data-dependent acquisition method mentioned above. The parameters of the mass spectrum were set as follows: MS1 scan resolution: 120000, AGC target:4e5, max injection time: 50 ms, mass range: 350 to 1250 m/z, followed by 40 data-independent acquisition scans with segment widths adjusted to the precursor density; MS2 scan resolution: 30 k, max injection time: 50 ms, AGC target: 5e5, HCD: 31%.

### Database search

MaxQuant (version 1.5.7.4) was used for data-dependent acquisition data search. The common search parameters: Type: standard, multiplicity 1; Digestion: digestion mode(specific), enzyme, trypsin/P; Variable modification: oxidation(M), acetyl (protein N-term); Max number of modifications per peptide: 5; Missed cleavage sites were allowed: 2; Label-free quantification: LFQ; LFQ minimum ratio count: 2; Fast LFQ was selected; LFQ minimum number of neighbors: 3; LFQ average number of neighbors: 6; Instrument: orbitrap; Fixed modification: carbamidomethyl (C); Two missed cleavage sites were allowed. We adopted the criteria for confident identification with a false discovery rate (FDR) < 0.01 at peptide and protein levels.

Data of data-independent acquisition searched by the direct DIA module of Spectronaut Pulsar (version 14.2.200619.47784). The common search parameters: Enzymes/Cleavage Rules: trypsin/P; XIC Extraction: default parameter; Modifications: Fixed modification: Carbamidomethyl (C); Variable modifications: Oxidation (M), Acetyl (Protein N-term); Calibration: default parameter; Identification FDR (false discovery rate) threshold: peptide levels: 0.01, protein levels: 0.01, and PSM levels: 0.01; Identification: Machine Learning: Per Run, Precursor, and protein Qvalue Cutoff: 0.01, Probability Cutoff: 0.75, and the others were default parameter; Quantification: Quantity MS-Level: MS2 and the others were default parameter; The Workflow, Post Analysis and Pipeline Mode parameter setting was default parameter.

The Database of Uniprot-Human-Filtered-Reviewed-Yes -UP000005640_9606.fasta was used for all database searches.

### RNA and protein quantification

For mRNA-seq and full-length translating mRNA-seq (RNC-seq) data, our study used the FANSe3 algorithms. The sequence mapping of FANSe3 can be referenced to the human transcriptome database. The mRNA abundance was normalized using RPKM.

For protein quantification analysis, label-free mass spectrometry data were quantified with the iBAQ (intensity-based absolute quantification) algorithm as provided in MaxQuant. Remove missing values from protein quantitative data before performing median normalization.

## Data Records

All the sequencing datasets are available at the NCBI Gene Expression Omnibus (GEO) with dataset identifier GSE234201^42^. All the mass spectrometry raw data are publicly available on iProX with the accession number PXD041292^43^. Details of all omics data are shown in supplementary Tables 2, 6.

## Technical Validation

### Quality control of cells

**Fig. 2:**
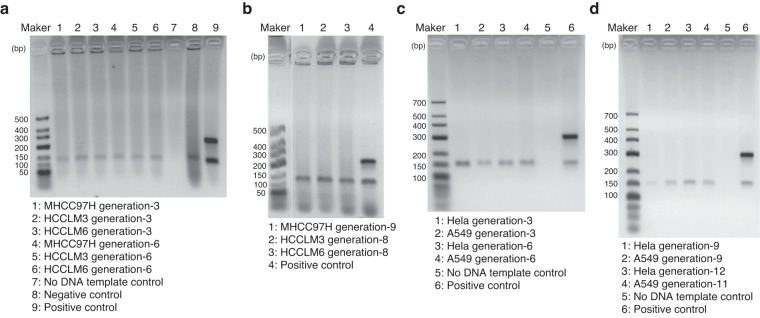
Results of mycoplasma detection in 5 cell lines. The marker used in (**a,b**) was DL500 DNA Marker (Takara, 3590Q, Japan), and the marker used in (**c,d**) was Low Ladder (Dongsheng Biotech, M1031, China). The concentration of agarose gel was 2% and the electrophoresis buffer was 1 × TBE. The electrophoresis conditions were: voltage: 100 V, current: 230 mA, electrophoresis time: 25~30 min. According to its user manual, the results of the cell samples were judged as follows: The DNA amplification product of the host cell was 150 bp band. When there were two bands of 280 bp and 150 bp, it represented mycoplasma positive. When there was only one 280 bp band, it represented mycoplasma contamination seriously. When there was only one 150 bp band, it was mycoplasma negative. When there was no band, the PCR reaction was inhibited.

To find a cell line with stable transcriptome and proteome during long-term subculture, we tested 5 commonly used cell lines: MHCC97H, HCCLM6, HCCLM3, Hela, and A549. In order to ensure the quality of cell lines, we detected mycoplasma at intervals to ensure that all cell lines were mycoplasma negative (Fig. 2a~d).

### RNA quality control

**Fig. 3:**
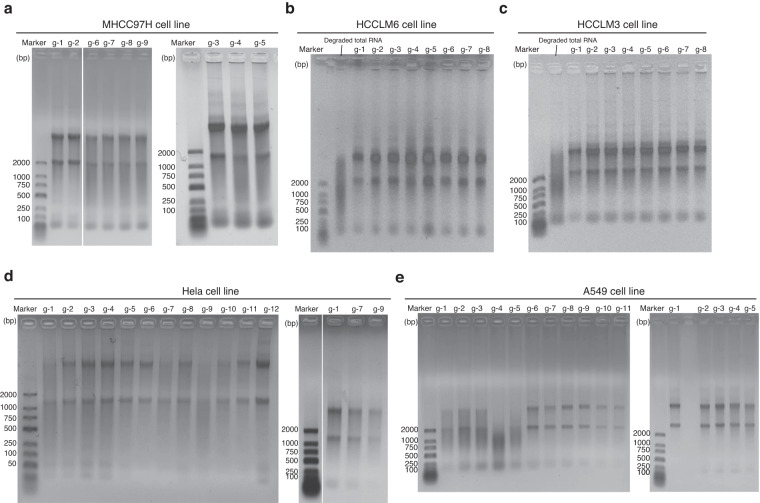
The intergrity test results of total RNA samples from 5 cell lines. The marker used in Fig. 3 was DL2000 DNA Marker (Takara, 3427 A, Japan). The concentration of agarose gel was 1% and the electrophoresis buffer was 1 × TBE. The electrophoresis conditions were: voltage: 100 V, current: 230 mA, electrophoresis time: 25~30 min. g-1, g-2, g-3, g-4, g-5, g-6, g-7, g-8, g-9, g-10, g-11 and g-12 represent the generation 1~12 of the cell line, respectively.

For each cell line, we cultured 8~12 generations and took samples from each generation. Total RNA was extracted from each sample and the RNA quality was examined by electrophoresis to verify that they were not degraded (Fig. 3a~e).

### Omics data quality control

We conducted quality control of sequencing data (including RNA-Seq and RNC-Seq) and generated a series of QC metrics. The overall quality of the sequencing dataset was satisfied at the level of raw and mapped data in the following aspects: (1) the average reads count of raw sequencing data was more than 20 M; (2) the average mapping ratio of all samples was around 72%; (3) the average GC content of the data generated from all samples was around 52%; (4) the average rRNA contamination ratio for all samples was around 4.57%; (5) the average Q30 of all samples was around 89%. The detailed results of data quality control are showed in supplementary Table 7.

### Reproducibility of transcriptome datasets

**Fig. 4:**
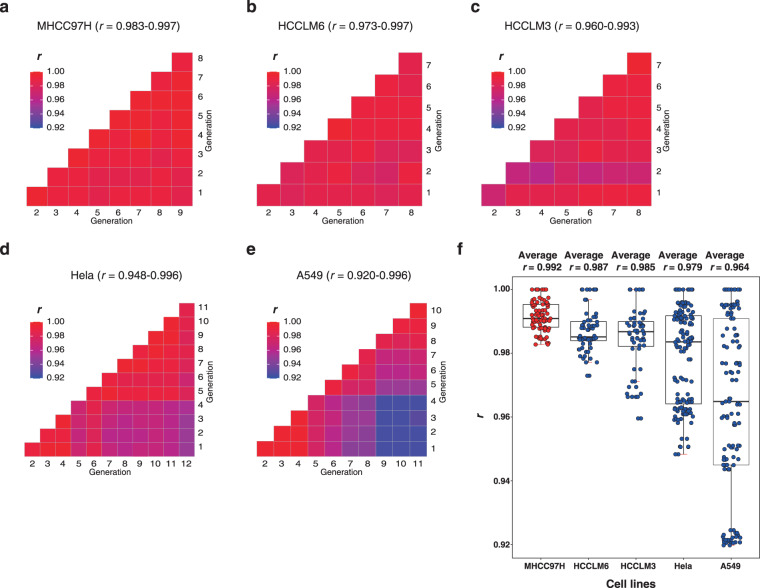
MHCC97H has the strongest transcriptome stability in the subculture of 5 cell lines. (**a**–**e**) Mutual Pearson correlation of the transcriptome of MHCC97H, HCCLM6, HCCLM3, Hela, and A549. (**f**) Distribution of the mutual Pearson correlation of the 5 cell lines. (**Note**: 1~12 represent generation 1~12, respectively. All correlation coefficients were statistically significant with *p* < 0.001).

We used the polyA + mRNA method to construct a transcriptome library for sequencing and used the RPKM method for quantification. The mutual correlation of gene expression showed that the MHCC97H has the most stable transcriptome, with the Pearson *r* = 0.983~0.997 (Fig. 4a). The other two hepatocellular carcinoma cell lines showed lower consistency (*r* can be as low as 0.973 and 0.960, respectively, Fig. 4b,c). The Hela and A549 cell showed even lower consistency over the generations (average *r* = 0.979 and 0.964, respectively, with the lowest value being 0.948 and 0.920, respectively, Fig. 4d~f).

**Fig. 5:**
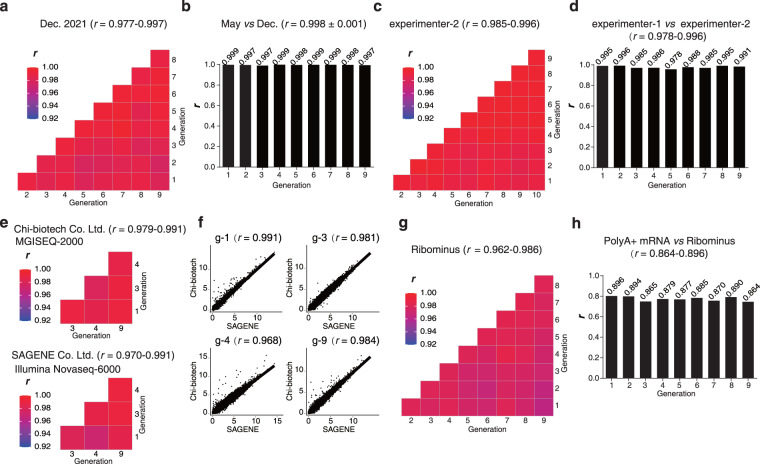
MHCC97H transcriptome was highly stable during subculturing. (**a**) The mutual Pearson correlation of the second batch of MHCC97H was subcultured in December 2021. The first batch shown in Fig. 4a was subcultured in May 2021. (**b**) The correlation of the same generation between the two batches of subcultured MHCC97H cell lines in May and December 2021 (comparing data shown in Figs. 4a and 5a). (**c**) The same series of total MHCC97H RNA was subjected to library construction by another experimenter using another batch of library construction kit. (**d**) Coincidence of the two experimenters and two batches of library construction kits (comparing data shown in Figs. 4a and 5c). (**e**) RNA samples from 4 randomly chosen generations (generation 1, 3, 4 and 9) of MHCC97H were distributed to two commercial sequencing service providers. Here shows the mutual Pearson correlation of the transcriptome quantification within each lab. (**f**) The cross-lab correlation of gene expression for each generation. (**g**) Similar to Fig. 4a, using the rRNA depletion method (Ribominus) to construct an RNA-seq library. (**h**) The correlation of gene expression between the PolyA + mRNA and Ribominus strategies for each generation. (**Note:** 1~10 represent the generation 1~10 of the cell line, respectively. All correlation coefficients were statistically significant with *p* < 0.001).

Subsequently, we performed a series of rigorous evaluations on the stability of MHCC97H at the transcriptome level. Firstly, we subcultured two batches of MHCC97H cell lines in May and December 2021, respectively. The mutual correlation of gene expression over generations was similar (*r* = 0.977~0.997, Fig. 5a). The correlation of the same generation between the two batches was steadily high (*r* = 0.998 ± 0.001, Fig. 5b). Secondly, we tested the robustness over experimenters and labs. The results (Fig. 5c~d) were almost identical to the former experimenter (Fig. 4a). We also sent 4 samples to two commercial sequencing service providers more than 1000 km away. Chi-Biotech Co. Ltd. was equipped with a MGISEQ-2000 platform, and Sagene Co. Ltd. was equipped with a NovaSeq-6000 platform. The Pearson *r* reached 0.979~0.991 and 0.970~0.991, respectively (Fig. 5e), and the mutual correlation of gene expression over generations was similar in both labs (Fig. 5f).

We then tested different mRNA enrichment strategies. Our standard protocol used oligo-dT to enrich polyA + mRNA (mature mRNA), which was applied in most studies. Another strategy was the rRNA depletion method, which removes rRNA by probe hybridization followed by beads extraction or RNase H degradation. Using the rRNA depletion method, the Pearson *r* = 0.962~0.986 (Fig. 5g), which was considerably lower than the oligo-dT method. As expected, the correlation of gene expression between the two strategies was slightly lower (*r* was only 0.864~0.896) (Fig. 5h), suggesting that the data generated by two different mRNA enrichment strategies should not be mixed for analysis.

**Fig. 6:**
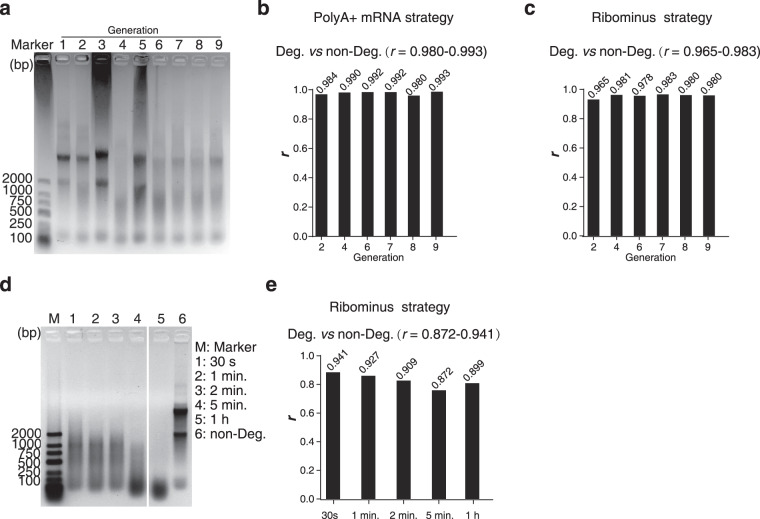
RNA-seq of degraded MHCC97H RNA samples. (**a**) The electrophoresis of the total RNA of MHCC97H which was frozen and thawed for 10 cycles and exposed to air for a prolonged time. (**b**) Correlation of the transcriptome quantification of the non-degraded and its corresponding degraded RNA samples by oligo-dT method. (**c**) Similar to Fig. 6b, but used the rRNA depletion method. (**d**) Artificially degraded MHCC97H total RNA by adding RNase A for 30 seconds to 1 hour. (**e**) Similar to Fig. 6c, for the RNaseA-degraded RNA. The marker used in Fig. 6 was DL2000 DNA Marker (Takara, 3427 A, Japan). The concentration of agarose gel was 1% and the electrophoresis buffer was 1 × TBE. The electrophoresis conditions were: voltage: 100 V, current: 230 mA, electrophoresis time: 25~30 min. (**Note:** 1~9 represent the generation 1~9 of the cell line, respectively).

The RNA is vulnerable to ubiquitous RNases and environmental changes (e.g. freeze-thaw cycles). Therefore, minor or major degradation might be inevitable during the production, storage, and transport of the standard samples. We generated transcriptome sequencing datasets of RNA-degraded samples to investigate how the degradation affects their applicability to serve as a reference. Firstly, we created a scenario that mimics the degradation due to environmental exposure: the RNA samples were exposed to the air at room temperature for a prolonged time (more than 2 hours), so that the RNases in the environment may enter the tube and degrade the RNA. Then, the samples were frozen and thawed for 10 cycles. The result of agarose gel electrophoresis showed that the total RNA of MHCC97H was degraded to various extents (Fig. 6a). Surprisingly, when we used oligo-dT method, the non-degraded RNA samples and their corresponding RNA degraded samples still had high transcriptome correlation (Pearson *r* = 0.980~0.993, Fig. 6b). The rRNA depletion method also showed remarkable consistency with the counterparts of non-degraded and degraded RNA samples (*r* = 0.965~0.983, Fig. 6c), but still lower than the oligo-dT method.

Most environmental RNases are exonucleases, which may remain the 3’-end of the mRNAs. However, endonucleases may degrade mRNA into smaller fragments. We added RNase A into the non-degraded MHCC97H total RNA and incubated for 30 seconds to 1 hour to create a series of endonuclease-degraded samples (Fig. 6d). However, the endonuclease-degraded samples showed considerably lower consistency compared to the non-degraded counterparts (*r* = 0.872~0.941, Fig. 6e), but still much higher than the correlation reported by other literature (*r*^*2*^ = 0.41~0.69)^13^.

### Reproducibility of translatomic and proteomic datasets

**Fig. 7:**
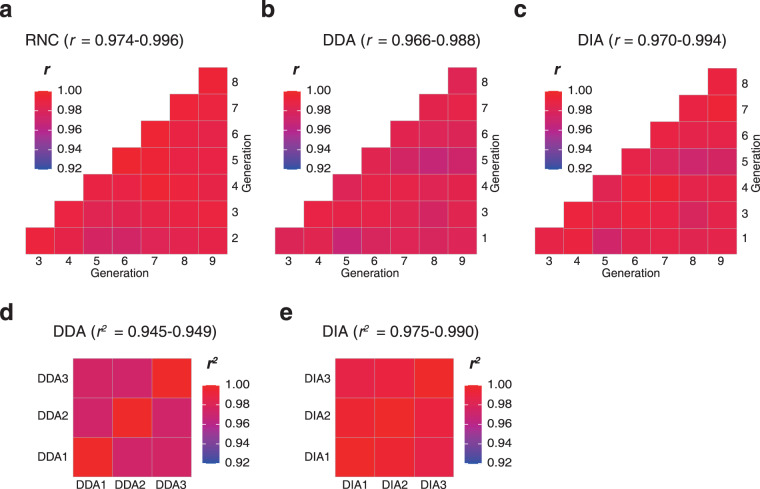
The stable translatome and proteome of MHCC97H. (**a**) The RNC-seq of the MHCC97H of 8 subculturing generations. (**b,c**) The consistency of protein abundance of the MHCC97H was detected by mass spectrometry in data-dependent acquisition (**b**) and data-independent acquisition (**c**) modes, respectively. (**d,e**) The technical triplicate of data-dependent acquisition (DDA) (**d**) and data-independent acquisition (DIA) (**e**) modes of one MHCC97H total protein sample. (**Note**: 1~9 represent the generation 1~9 of the cell line, respectively. All correlation coefficients were statistically significant with *p* < 0.001).

It is generally known that translational regulation is the most significant regulatory level^22^. Therefore, we first tested the stability of the MHCC97H translatome over subculture generations. The RNC-seq of the MHCC97H showed very high mutual consistency (Pearson *r* = 0.974~0.996, Fig. 7a). Mass spectrometry requires more steps than RNA-seq, making it difficult to achieve high reproducibility. However, the protein abundance detected using mass spectrometry was comparable both in data-dependent acquisition mode (*r = *0.966~0.988) (Fig. 7b) and in data-independent acquisition mode (*r* = 0.970~0.994) (Fig. 7c), respectively. To test the variability contributed by the experimental procedures, we started from the same trypsin-digested sample and made 3 independent mass spectrometry measurements (including LC-MS and data analysis). Such technical replicates yielded *r*^*2*^ = 0.945~0.949 and *r*^*2*^ = 0.975~0.990 in data-dependent acquisition (Fig. 7d) and data-independent acquisition (Fig. 7e) modes, respectively. These results indicated that the variance contributed by biological nature and trypsin digestion could be neglected in the DDA mode, and merely distinguishable in the DIA mode.

**Fig. 8:**
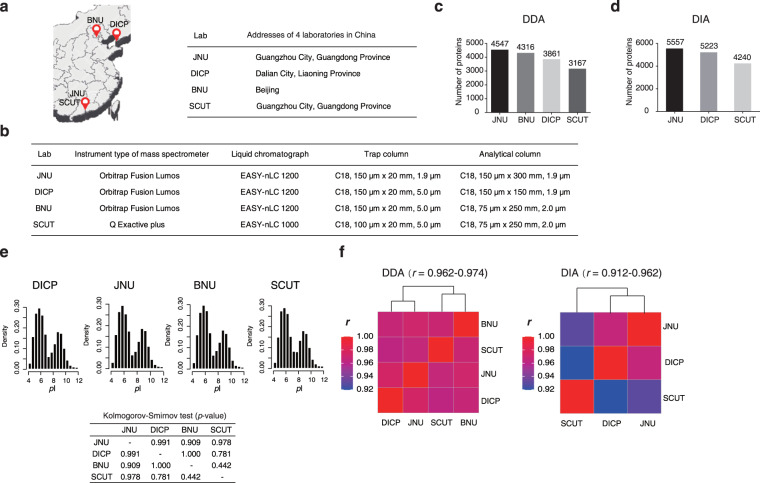
The repeatability of the MHCC97H proteome was verified in several laboratories. (**a**) The locations of the four laboratories in China (JNU, SCUT, DICP, and BNU). (**b**) The hardware configuration of mass spectrometry instruments in 4 labs (JNU, SCUT, DICP, and BNU). (**c**) The number of proteins in different laboratories in data-dependent acquisition modes. (**d**) The number of proteins in different laboratories in data-independent acquisition modes. (**e**) Distribution of isoelectric point of the same batch of MHCC97H protein samples identified by 4 laboratories, and their mutual Kolmogorov-Smirnov tests. (**f**) Mutual Pearson correlation of the protein abundances measured by 4 laboratories in the data-dependent acquisition and data-independent acquisition modes, respectively. (All correlation coefficients were statistically significant with *p* < 0.001).

Next, we tested the robustness of the standard proteome sample across labs and instruments. We distributed the same batch of standard proteome samples to 4 labs (JNU, SCUT, DICP, and BNU), which were over 2000 km away (Fig. 8a). The samples were shipped using ice boxes at 0 °C for 3 days. All labs followed the same protocol to process the samples. The only hardware differences were listed in Fig. 8b. A similar number of protein were identified in the 4 labs (Fig. 8c~d). The JNU lab yielded slightly more proteins due to the long column, which provides higher chromatography resolution. The SCUT lab yielded fewer proteins due to the lower resolution and slower scanning speed of the mass spectrometer. However, the distribution of the isoelectric points (*p*I) of the identified protein showed no significant differences among these labs (Fig. 8e). The protein abundance measured by these labs was highly comparable (*r = *0.962~0.974, Fig. 8f left). In data-independent acquisition mode, the labs with the same instruments showed highly similar results (*r* = 0.962), while the SCUT lab, which was equipped with another model of mass spectrometer showed remarkably lower consistency to the other two labs (*r* = 0.912) (Fig. 8f right), demonstrating that the instrument-specific bias and spectrum analysis software cannot be neglected.

## Supplementary information

Supplementary Table 2

Supplementary Table 3

Supplementary Table 4

Supplementary Table 5

Supplementary Table 6

Supplementary Table 7

Supplementary Table 1

## Data Availability

The data analysis methods, software, and associated parameters used in the present study were described in the section of Methods. If no detailed parameters were described for the software used in this study, default parameters were employed. No custom scripts were generated in this work.
